# Luteolin alleviates ulcerative colitis in rats via regulating immune response, oxidative stress, and metabolic profiling

**DOI:** 10.1515/med-2023-0785

**Published:** 2023-08-30

**Authors:** Bolin Li, Yuxi Guo, Xuemei Jia, Yanru Cai, Yunfeng Zhang, Qian Yang

**Affiliations:** Department of Gastroenterology, Hebei Provincial Hospital of Chinese Medicine, Shijiazhuang, Hebei, China; Key Laboratory of Integrated Chinese and Western Medicine for Gastroenterology Research (Hebei), Shijiazhuang, Hebei, China; Graduate School, Hebei University of Traditional Chinese Medicine, Shijiazhuang, Hebei, China; Hebei Key Laboratory of Turbidity Toxin Syndrome, Shijiazhuang, Hebei, China; Department of Gastroenterology, Hebei Provincial Hospital of Chinese Medicine, 389 Zhongshan East Road, Chang’an District, Shijiazhuang, Hebei, China

**Keywords:** ulcerative colitis, luteolin, oxidative stress, immune response, metabolomics, liquid chromatography-mass spectrometry

## Abstract

Ulcerative colitis (UC) is an inflammatory bowel disease and associated with metabolic imbalance. Luteolin (LUT) reportedly exhibits anti-inflammatory activity. However, its regulatory effects on metabolites remain indistinct. Here, the effects of LUT on immune response and oxidative stress in UC were determined. Serum metabolomics profiles of UC rats treated with LUT were obtained utilizing liquid chromatography-mass spectrometry. The results revealed that LUT treatment alleviated colon tissue injury, colon shortening, weight loss, and inflammatory response in UC rats. Additionally, the levels of superoxide dismutase and total antioxidant capacity were elevated, but malondialdehyde content was reduced in serum of UC rats, while these changes were abrogated by LUT. Metabolomics analysis unveiled that l-malic acid, creatinine, l-glutamine, and l-lactic acid levels were remarkably decreased, while dimethyl sulfone, 5-methylcytosine, cysteine-S-sulfate, and jasmonic acid levels were notably increased after LUT treatment. Furthermore, differential metabolites primarily participated in d-glutamine and d-glutamate metabolism, glutathione metabolism, and citrate cycle pathways. In summary, these results demonstrated that LUT improved immune response, alleviated oxidative stress, and altered metabolites in UC rats. This study lays the root for further exploring the mechanism of LUT in the treatment of UC.

## 1 Introduction

Ulcerative colitis (UC) is a chronic inflammatory disease that influences the colon, and usually presents with abdominal pain and bloody diarrhea [1]. The pathogenesis of UC includes multiple factors such as heredity, epithelial barrier defect, immune response disorder, and environment [2]. At present, the incidence of UC is on the rise in the world [3]. Approximately 95% of cases involve the rectum and develop continuously in a symmetrical, circumferential pattern to the vicinity, involving part or all of the large intestine [4]. Up to 15% of patients with UC require colectomy [2]. The common approaches for treating UC mainly included antibiotics, 5-aminosalicylates, glucocorticoids, and immune-mediators [5,6]. However, these drugs exert numerous limitations, including adverse side effects and economic burden. Thus, it is necessary to find a novel treatment which overcomes the shortcomings of the current treatments.

Luteolin (LUT) is a natural flavonoid compound and is widely found in many plants, including pepper, carrot, chamomile, celery, and spinach [7,8]. LUT has been reported to have antioxidant, antiviral, anti-inflammatory and anti-cancer properties [9]. In the anti-inflammatory studies of LUT, LUT was found to reduce the damage of bronchopneumonia, inhibit the inflammasome, reduce neutrophilic inflammation, treat osteoarthritis, reduce the inflammatory phenotype of high-glucose induced cardiomyocytes, and prevent non-alcoholic steatohepatitis [10–15]. Evidence revealed that LUT has a protective effect against UC [16]. However, the therapeutic mechanism of LUT in UC has not been fully elucidated.

In recent years, metabolomics has been developed rapidly. Metabolomics analysis can significantly improve the chance of finding disease-related biomarkers [17]. Zhang et al. compared UC patients with controls and found that UC patients showed increased 3-hydroxyl, β-glucose, d-glucose, and phenylalanine, but decreased serum lipids [18]. Bjerrum et al. found that leucine, isoleucine, valine, and lysine increased in feces of UC patients [19]. These findings indicated that metabolites were associated with the development UC. Liquid chromatography-mass spectrometry (LC-MS) has been widely used in recent years because of its advantages of high sensitivity, strong specificity, and simple sample treatment [20]. Metabolomics analysis of urine and feces sample in UC rats by using LC-MS revealed that Berberine could improve metabolic disorders [21]. However, the profiles and differences of serum metabolites of UC rats regulated by LUT have not been reported.

In this study, we aimed to explore the function of LUT in immune response, oxidative stress, and metabolites in UC rats. UC model was established by employing dextran sulfate sodium (DSS) administration in rats. The metabolic profiles and potential metabolites in the UC rats after LUT treatment were investigated by using LC-MS. This study offers a basis for the potential metabolic mechanism of LUT in UC.

## 2 Materials and methods

### 2.1 Animals

Eight-week-old male Wistar rats (200 ± 20 g) were obtained from Hebei Provincial Hospital of Chinese Medicine (Animal number: SCXK(Ji)2018-004). All rats were grown in a specific pathogen-free room with 40–50% humidity and 12 h/12 h dark/light cycle at 22–24°C. They had free access to food and water *ad libitum*. All rats were adapted to environment for a week before experiment.

### 2.2 Induction of UC and animal treatment

The UC rat model was induced by DSS (Dalian Meilun Biotechnology Co., LTD, China). The rats were randomly segmented into four groups (*n* = 10 each group): normal control group (NC group), model group (MOD group), mesalazine (MES) group, and LUT group. MES was used as a positive control. The rats of the NC group were given drinking water. The rats of the MOD, MES, and LUT groups were given 3.5% DSS solution for continuous 10 days to build UC model rats [22]. After the UC model was successfully established, rats in the MES group were administrated intragastrically with MES suspension (Losan Pharma GmbH, Freiburg im Breisgau, Germany) at a usage of 0.315 g/kg/day once daily for 14 days based on our preliminary experiment. Rats in the LUT group were administrated intragastrically with LUT suspension (Shanghai Winherb Medical Technology Co., Ltd, China) at usage of 34.6 mg/kg/day once daily for 14 days based on our preliminary experiment. Rats in the NC group and MOD group were given the same dose of normal sodium chloride. Fourteen days after treatment, the rats were fasted for 24 h. No rats died in the NC group and the LUT group. One rat died in the MOD group and one rat died in the MES group during the treatment. Then rats were injected with 1% pentobarbital sodium (50 mg/kg) intraperitoneally. All rats were sacrificed at the end of experiment for collecting colon tissues and serum. The samples were stored at −80°C until use.

### 2.3 Enzyme-linked immunosorbent assay (ELISA)

The levels of TNF-α, C-reactive protein (CRP), IL-13, IL-33, and suppression of tumorigenicity 2 (ST2) in the serum were measured using corresponding ELISA kits (Boster Biological Technology Co. Ltd, USA) in accordance with the instructions from the manufacturer.

### 2.4 Hematoxylin–eosin (H&E) staining

The pathological changes in colon tissues of rats were evaluated by H&E staining. Briefly, colon tissues were fixed in 10% buffered formaldehyde (Shanghai Biotechnology Co. Ltd, Shanghai, China), dehydrated, and embedded in paraffin. The colon segments was sliced with a thickness of 4 μm, dewaxed in xylene, and rehydrated. Subsequently, the sections were stained with H&E. In the end, the sections were dehydrated in gradient ethanol, vitrified by xylene, and sealed with neutral balsam. The images were observed utilizing a light microscope (LEICA DM500, Wetzlar, Germany).

### 2.5 Immunohistochemistry

The paraffin sections were deparaffinized, rehydrated in alcohol. After soaking in 3% H_2_O_2_, the sections were boiled in 0.01 M sodium citrate buffer for antigen retrieval. The sections were blocked with 5% BSA and incubated with primary antibody against CD4 (1:2,000; Abcam, UK) and CD8 (1:2,000; Abcam, UK) overnight at 4°C. After washing four times with PBS, a secondary antibody (1:2,000; Abcam, UK) was added and the sections were incubated for 35 min at 37°C. In the end, the sections were stained with 3,3′-diaminobenzidine tetrahydrochloride and hematoxylin. The samples were dehydrated and mounted. Images were observed by employing a microscope (OLYMPUS CK31, Japan).

### 2.6 Assessment of oxidative stress

The level of superoxide dismutase (SOD), malondialdehyde (MDA), and total antioxidant capacity (T-AOC) in the serum of rats was measured employing SOD (ml059387-1, Shanghai EnzymeLinked Biotechnology, China), MDA (S0131S, Beyotime, China), and T-AOC (S0121, Beyotime, China) assay kits. After detecting the absorbance at 450 nm, the SOD, MDA, and T-AOC content was calculated following the manufacturer’s instructions.

### 2.7 Metabolite extraction and LC-MS

Samples were melted at 4°C. After 100 µL of each sample was transferred into 2 mL centrifuge tubes and added with 100 µL of mixed internal standard solution and 400 µL of methanol, the tubes were vortexed for 1 min. Subsequently, the samples were centrifuged at 4°C for 10 min at 12,000 rpm, and 500 µL of the supernatant was transferred into a new 2 mL centrifuge tube. Samples were concentrated to dry and dissolved with 150 µL of 80% methanol solution. Finally, centrifugation was performed to obtain the supernatant for LC-MS.

Chromatography was carried out in an Thermo Vanquish (Thermo, MA, USA) system, which had an ACQUITY UPLC^®^ HSS T3 (150 × 2.1 mm, 1.8 µm, waters) column. The temperature of the autosampler and column was 8 and 40°C, respectively. Gradient elution of samples was conducted with 0.1% formic acid in water and 0.1% formic acid acetonitrile or 5 mM ammonium formate in water and acetonitrile.

The ESI-MSn experiments were performed on the Thermo Q Exactive mass spectrometer (Massachusetts, USA). MS parameters were set as follows: the spray voltage was 3.5 kV in positive modes and 2.5 kV in negative modes. The capillary temperature was 325°C. The scanned range was *m*/*z* 81–1,000 with a resolution ratio of 70,000. Data were acquired in data-dependent acquisition mode or MS/MS mode to get detailed information of the compounds.

### 2.8 Metabolite identification and metabolic pathway enrichment analysis

The raw data were transformed into mzXML format through Proteowizard software (v3.0.8789). R package (v3.3.2) was employed for peaks identification, peaks filtration, and peaks alignment. Mass to charge ratio, retention time, and intensity were obtained and batch normalization of these data was conducted. Afterward, quality control and quality assurance were carried out to obtain high-quality metabolomics data. Partial least-squares discriminant analysis (PLS-DA) was utilized to determine the altered compounds of all samples. The relevant differential metabolite screening criteria were *P*-value ≤ 0.05 and VIP ≥ 1. The identification of metabolites was first determined following the precise molecular weight, and then annotated with Metlin database and MoNA database. MetaboAnalyst and Kyoto Encyclopedia of Genes and Genomes (KEGG) were utilized to analyze the metabolic pathways.

### 2.9 Statistical analysis

SPSS 19.0 was utilized to analyze data and data were displayed as mean ± standard deviation (SD). The one-way analysis of variance followed by Tukey’s *post hoc* test was selected to detect the statistical significance of more than two groups. *P*-value less than 0.05 was deemed to be statistically significant.


**Ethics statement:** All rat’s experiments were approved by the Ethical Committee and the Animal Experimental Committee of Hebei Provincial Hospital of Chinese Medicine.

## 3 Results

### 3.1 LUT protects DSS-induced colitis rats from colonic damage

**Figure 1:**
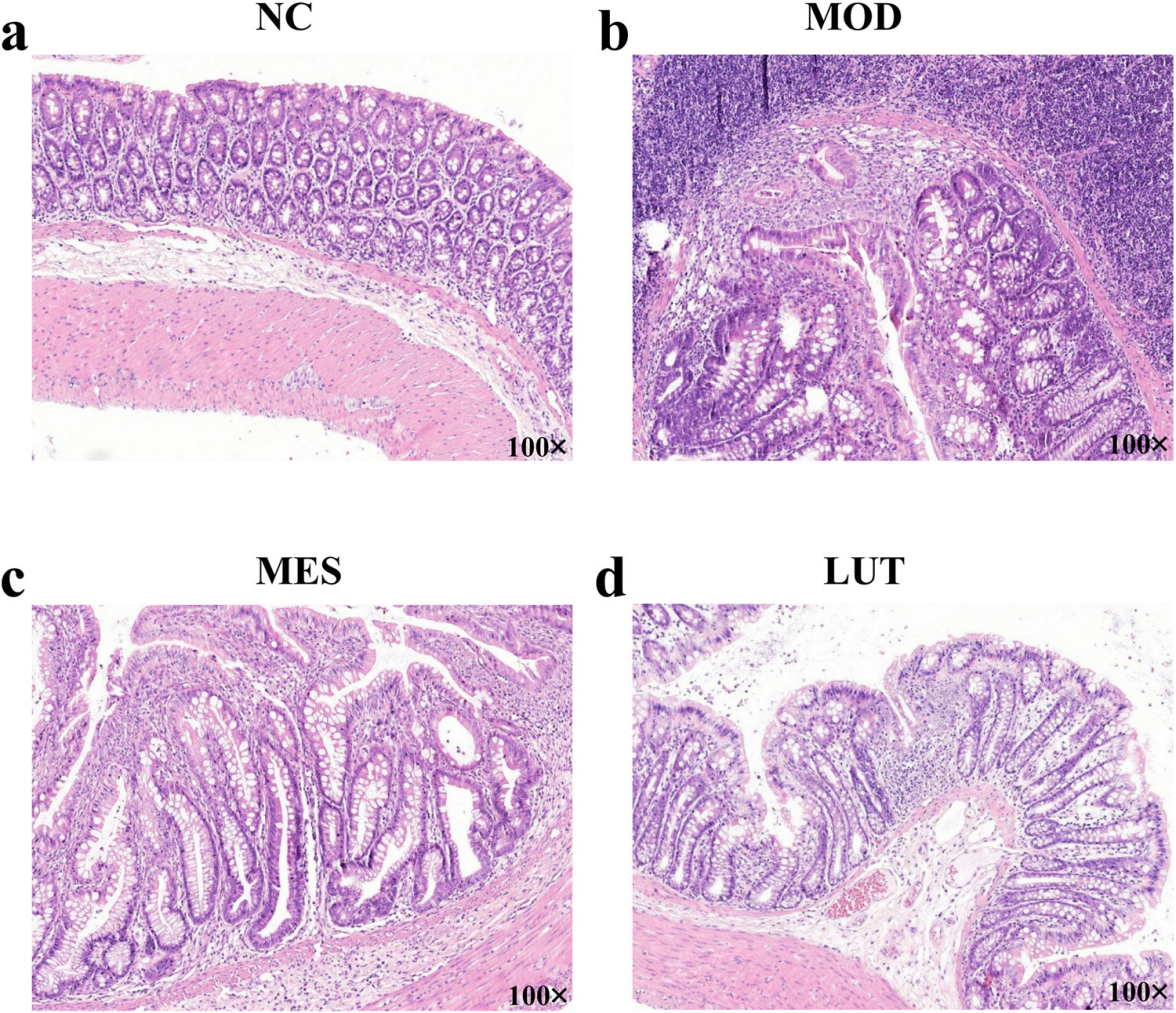
Effects of LUT on the histological changes in UC rats. Histological changes of rats in (a) normal control (NC), (b) model (MOD), (c) mesalazine (MES), and (d) luteolin (LUT) group were observed by HE staining (100× magnification).

To ascertain the function of LUT in histological changes of UC rats, the colon tissues of rats were observed. As displayed in Figure S1a, the appearance of colorectal was normal in the NC group. Compared with the NC group, the colorectal length in the MOD group was shortened, the intestinal cavity was filled with dark red feces, and part of the intestinal wall was thinner (Figure S1b). The colorectal lengths of rats in the MES group and the LUT group were improved (Figure S1c and d). The UC rats demonstrated decreased body weight compared with the normal rats. The administration of MES or LUT resulted in an increase in body weight in UC rats (Table S1). HE staining indicated that the structure the colon of rats was intact and clear without inflammation and ulcer in the NC group. The glands were arranged regularly (Figure 1a). In the MOD group, the colonic epithelial structure, crypt structure, and epithelial integrity of rats were destroyed. The glands are badly damaged with submucosal edema and a large number of inflammatory cell infiltration (Figure 1b). After treatment with either MES or LUT, the colonic mucosal epithelium of rats was basically intact, the edema and destruction of crypt were alleviated, and inflammatory cell infiltration was obviously reduced in UC rats (Figure 1c and d). These data demonstrated that LUT could ease colonic damage of UC rats.

### 3.2 LUT improves immune response in the UC rats

**Figure 2:**
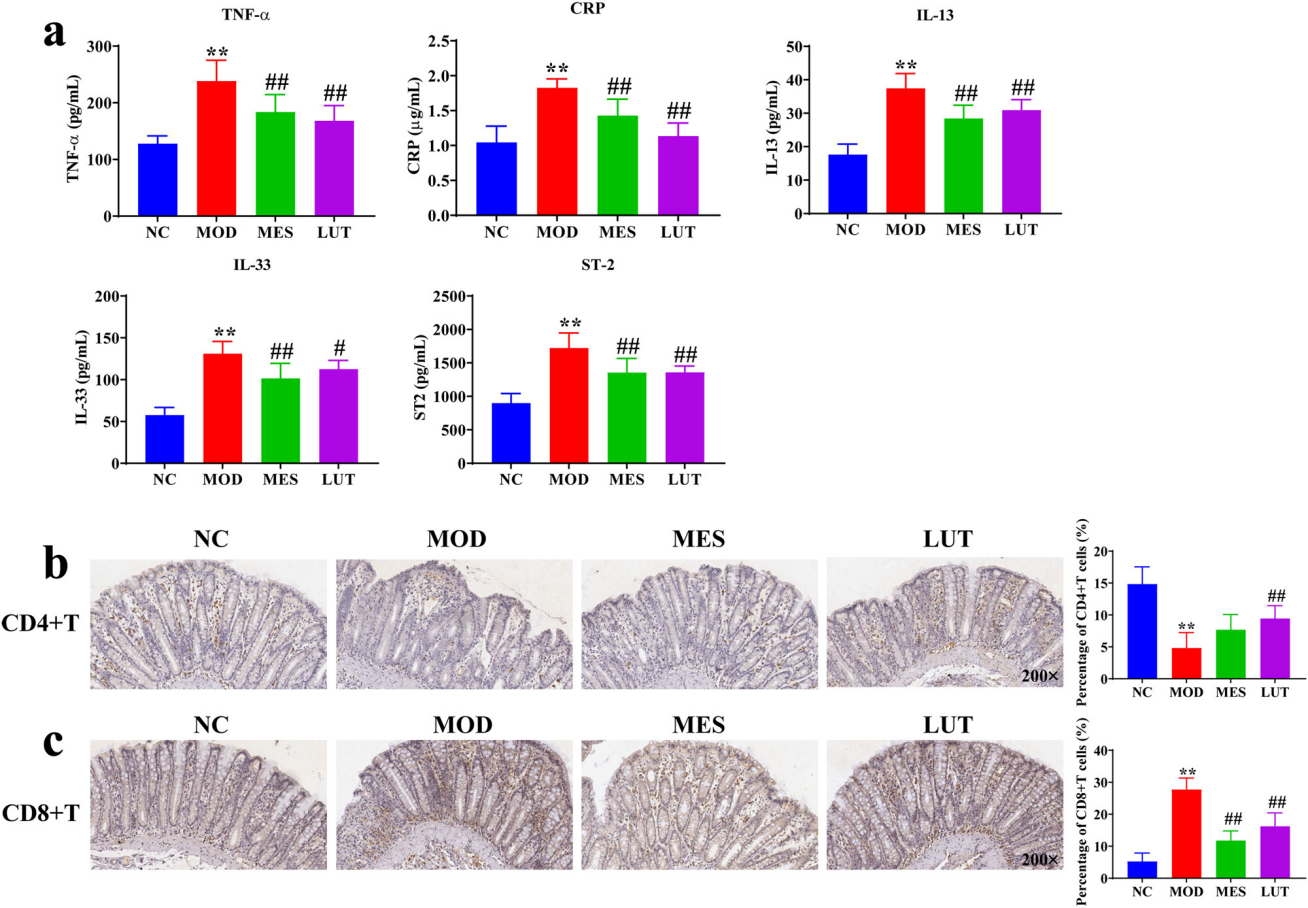
Effects of LUT on inflammation and immune response in UC rats. (a) Expression levels of TNF-α, CRP, IL-13, IL-33, and ST-2 were examined by ELISA. (b) Representative image of CD4+ T cell staining in the colon (100× magnification). (c) Representative image of CD8+ T cell staining (100× magnification). NC represents normal control. MOD represents model. MES represent mesalazine. LUT represents luteolin. There were 10, 8, 8, and 9 replicates in NC, MOD, MES, and LUT groups, respectively. **P < 0.01 vs the NC group. #*P* < 0.05 and ##*P* < 0.01 vs the MOD group.

To investigate the effects of LUT on inflammation, we examined the levels of inflammatory factors, including TNF-α, CRP, IL-13, IL-33, and ST-2 by ELISA. In comparison with the NC group, the levels of TNF-α, CRP, IL-13, IL-33, and ST-2 were remarkably elevated in the MOD group. Nevertheless, LUT administration prominently lowered these inflammatory factors in UC rats (Figure 2a). In addition, the immunohistochemistry was used to detect the levels of CD4+ T and CD8+ T cells. The proportion of CD8+ T cells was strikingly increased, whereas CD4+ T cells was markedly decreased in UC rat relative to the NC group. In contrast, LUT administration resulted in a prominent reduction in the ratio of CD8+ T cells and a dramatical increase in the ratio of CD4+ T cells (Figure 2b and c). Collectively, these results uncovered that LUT improves immune response in UC rats.

### 3.3 LUT inhibits oxidative stress in UC rats

**Figure 3:**
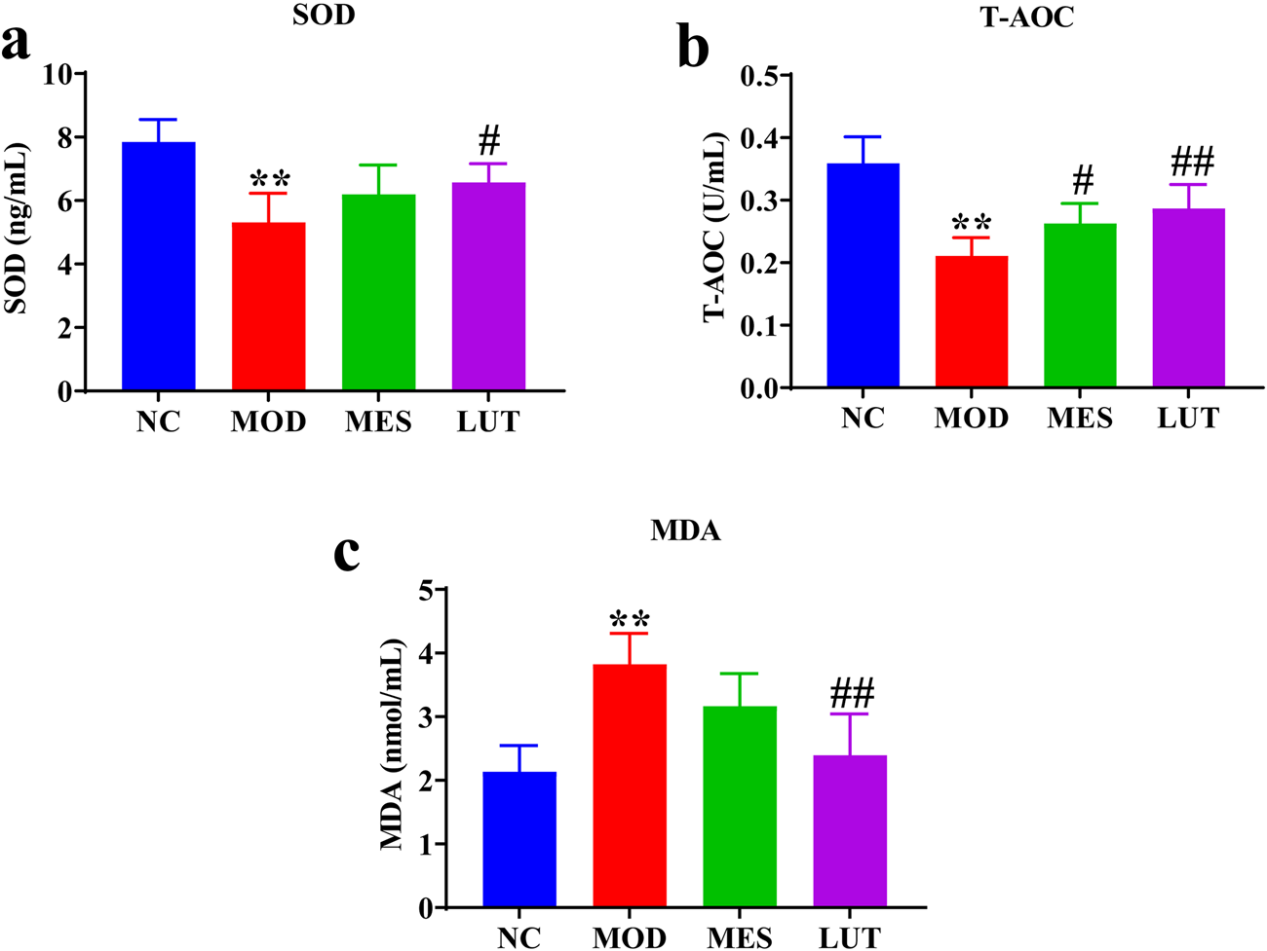
Effects of LUT on oxidative stress in UC rats. The levels of (a) SOD, (b) MDA, and (c) T-AOC in serum were detected by SOD, MDA, and T-AOC assay kits, respectively. Results are shown as the mean ± SD. NC represents normal control. MOD represents model. MES represent mesalazine. LUT represents luteolin. There were 10, 8, 8, and 9 replicates in NC, MOD, MES, and LUT groups, respectively. **P < 0.01 vs NC group. #*P* < 0.05 and ##*P* < 0.01 vs the MOD group.

We assessed the function of LUT in oxidative stress in UC rats. As displayed in Figure 3, the levels of SOD and T-AOC were obviously decreased and the MDA concentration was remarkably increased in UC rats relative to the NC group. On the contrary, their expressions were reversed by LUT treatment. These results indicated that LUT alleviated oxidative stress in UC rats.

### 3.4 Analysis of metabolic profiles of LUT-treated UC rats

**Figure 4:**
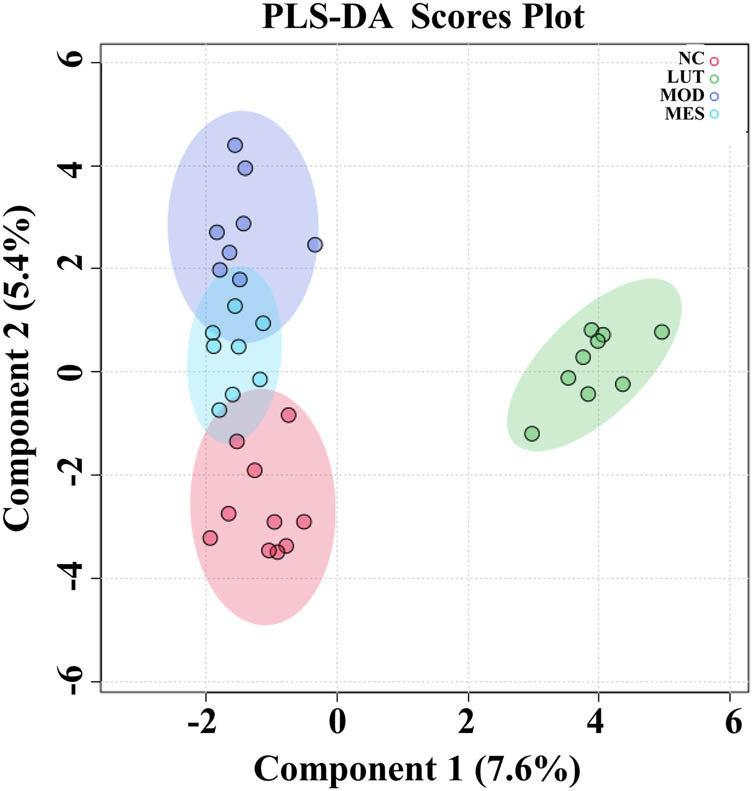
PLS-DA of serum metabolic profiling between normal control (NC), model (MOD), mesalazine (MES), and luteolin (LUT) groups.

To acquire credible and high-quality metabolomic data, the quality control and assurance were performed. The PCA score plots revealed that the samples clustered together (Figure S2a and b), indicating that the data are reliable. Additionally, 82.9% of ions in negative ion mode and 74% of ions in positive ion mode exhibited less than 30% of relative SD, which indicated that the reproducibility of the metabolomics method was excellent. Subsequently, to study the metabolic profiling changes of LUT treatment in UC, PLS-DA was conducted. According to the PLS-DA score plot, the metabolic profiles of the MOD group were far from the NC group (Figure 4). In addition, the metabolic profiles of the LUT group were far from those of the MOD group. Moreover, the metabolic profiles of the LUT group were also far from those of the MES group. These results indicated that metabolites altered between every two of all the groups.

### 3.5 Identification of potential metabolites

**Figure 5:**
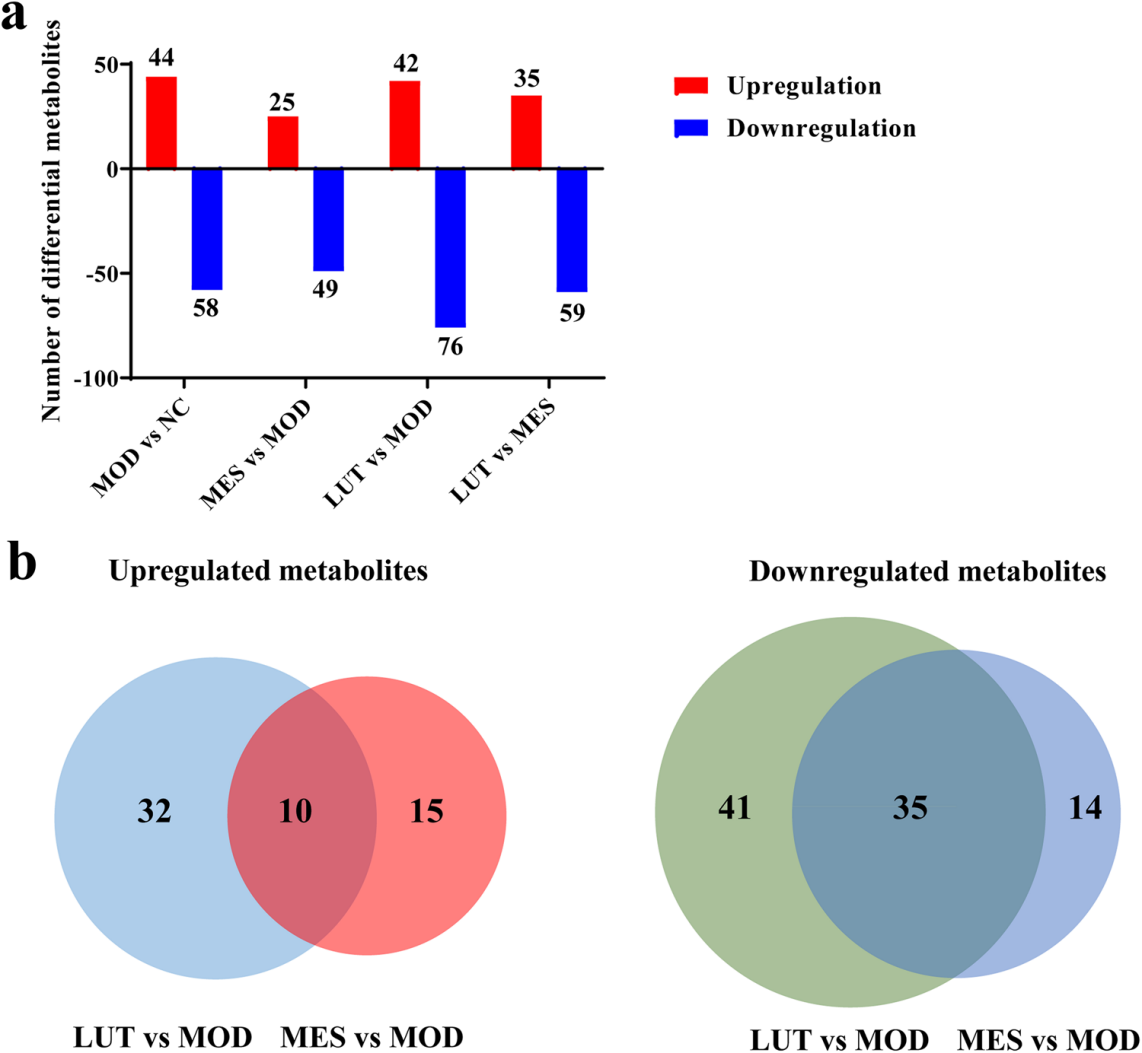
Analysis of differential metabolites in serum of UC rats. (a) Number of differential upregulated and downregulated metabolites in MOD vs NC, MES vs MOD, LUT vs MOD, and LUT vs MES. (b) Venn diagram showing common and differential upregulated and downregulated metabolites between LUT vs MOD and MES vs MOD. NC represents normal control. MOD represents model. MES represents mesalazine. LUT represents luteolin.

Differential metabolites were screened based on the standard with VIP > 1 and *p* < 0.05. We discovered that 102, 74, 118, and 94 metabolites were significantly altered in MOD vs NC, MES vs MOD, LUT vs MOD, and LUT vs MES, respectively (Figure 5a). Interestingly, there were ten common upregulated metabolites and 35 common downregulated metabolites between LUT vs MOD and MES vs MOD (Figure 5b). In addition, a total of 31 differential metabolites were significantly reversed after LUT was used to treat UC in rats (Table S2). In detail, in contrast with the NC group, the levels of 20 metabolites were notably increased in the MOD group, mainly including pimelic acid, deoxyuridine, carnosine, creatinine, phenylacetic acid, gluconic acid, dethiobiotin, indoleglycerol phosphate, isocitric acid, l-glutamine, l-malic acid, and l-lactic acid, and 11 metabolites were notably decreased, mainly including dimethyl sulfone, 5-methylcytosine, cysteine-S-sulfate, homovanillin, arachidic acid, androsterone, and jasmonic acid. However, after LUT treatment, the trend of these metabolites was prominently reversed (Table S2). Similarly, studies have reported that some metabolites such as malic acid, glutamine, lactic acid, and creatinine were associated with the development of UC [23–26]. Therefore, LUT might be involved in the treatment of UC via these metabolites.

### 3.6 Metabolic pathway analysis

**Figure 6:**
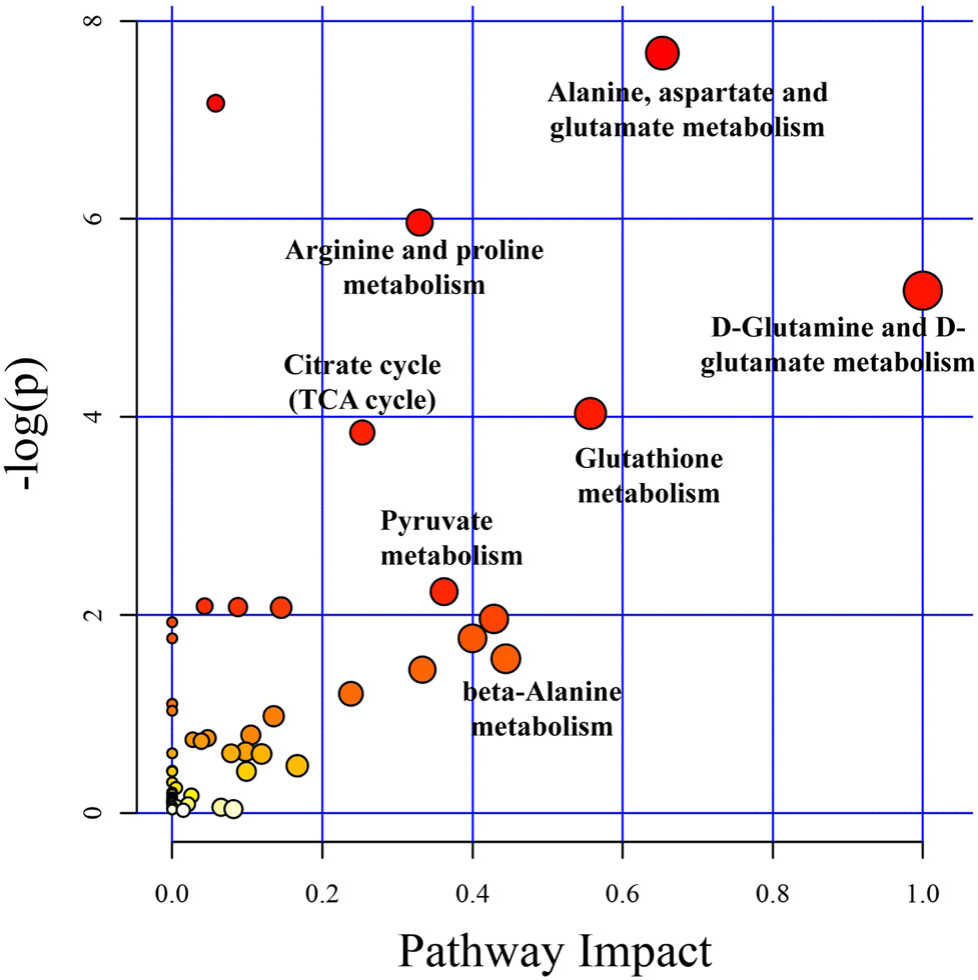
Analysis of metabolic pathway in serum of UC rats treated with LUT. Each point represents one metabolic pathway. The size of the dots indicates the impact on the metabolic pathways.

Metabolic pathway analysis between MOD and LUT groups was carried out according to the KEGG database and MetPA database. The significant metabolic pathways associated with LUT treatment mainly included d-glutamine and d-glutamate metabolism, alanine, aspartate and glutamate metabolism, citrate cycle (TCA cycle), arginine and proline metabolism, glutathione metabolism, pyruvate metabolism, and beta-alanine metabolism (Figure 6; Table S3). Among these pathways, d-glutamine and d-glutamate metabolism, glutathione metabolism, and citrate cycle pathways were associated with the development of UC, which have been reported to be involved in inflammatory response [27–29].

## 4 Discussion

UC is a chronic inflammatory disease characterized by mucosal inflammation, diarrhea, abdominal pain, and hematochezia [30]. Our results demonstrate that levels of TNF-α, CRP, IL-13, IL-33, and ST-2 were prominently elevated in UC rats. However, the opposite results were observed in UC rats treated with LUT. IL-13 is a cytokine which is released by Th2 cells, contributes to the pathology of UC, and participates in immune-mediated processes [31]. IL-33, belonging to the IL-1 cytokine family, exerts a vital role in various inflammatory bowel diseases including UC [32]. A previous study discovered that baicalin can alleviate UC via suppressing the expression of IL-33 [33]. Collectively, these results suggest that LUT alleviates inflammatory response in UC rats.

The imbalance of immune regulation is an important cause of UC [34]. As the central link of immune regulation, T lymphocytes exert a crucial role in the development of UC. Among them, CD4+ T cells and CD8+ T cells are closely relevant to the pathogenesis of UC [35,36]. Extract from mango mistletoes *Dendrophthoe pentandra* alleviates trinitrobenzene sulfonic acid-induced colitis via modulating CD4+ T cells [37]. The exogenous angiopoietin-like 4 protein can mitigate DSS-induced colitis through reducing CD8+ T cells in the colon [38]. In agreement with these studies, we found that LUT enhanced the ratio of CD4+ T cells and reduced the ratio of CD8+ T cells in UC rats. These findings unveil that CD4+ T cells and CD8+ T cells are crucial in the protective effect of LUT on UC.

Oxidative stress is a key factor in the occurrence of UC. It has been reported that MDA content as indicators of oxidative injury could cause serious cell damage and was enhanced in the colon tissues during UC [39]. SOD as an important endogenous antioxidants protects tissues from damage caused by reactive oxygen species [40]. It was reported that the level of MDA was increased but SOD was decreased in UC mice, while deoxyschizandrin inhibited the effects of DSS on the two levels [41]. A recent study discovered that 5-hydroxy-4-methoxycanthin-6-one reduced the level of MDA and enhanced the levels of SOD in DSS-induced colitic rats [42]. In line with these above studies, our data showed that SOD and T-AOC levels were decreased, while MDA concentration was increased in UC rats. However, their levels were reversed by LUT treatment. These results demonstrate that LUT may inhibit oxidative stress of UC rats and thus reduce the oxidative stress-induced tissues damage.

In this study, to explore the potential therapeutic mechanism of LUT in UC, we focused on the alteration of metabolic profiles in UC rats. We discovered that after DSS treatment in UC rats, amino acid metabolism changed significantly, leading to an upregulation of l-glutamine, and differential metabolites were principally implicated in d-glutamine and d-glutamate metabolism. Studies have reported that glutamine functions in inflammatory response, oxidative stress, cell protection, and intestinal barrier [43–45]. In addition, glutamine supplementation can improve UC [24]. Jeong et al. reported that glutamine exerted anti-inflammatory activity and improved DSS-induced colitis through MKP-1 induction [46]. Ren et al. also found that l-glutamine supplementation had beneficial effects on DSS-induced colitis [47]. Therefore, LUT might ease the inflammation in UC by regulating d-glutamine and d-glutamate metabolism.

Citrate cycle is the most efficient way to acquire energy and offers raw materials for the biosynthesis of numerous important substances [48]. A previous study reported that downregulation of citrate cycle could result in the decrease of energy supply in the colitis [49]. Another study performed metabolomics analysis of UC rats treated with Berberine and found that the metabolites in citrate cycle were remarkably altered by DSS administration and adjusted by Berberine treatment [14]. In our study, citrate cycle was the main metabolic pathway, which was related to LUT treatment. During the citrate cycle, the levels of pyruvic acid and fumaric acid were notably diminished in UC rats after LUT treatment. These data manifest that LUT can regulate the metabolic imbalance in citrate cycle in UC rats.

In conclusion, our study found that LUT attenuated colonic damage, and improved immune response and oxidative stress of UC rats. d-Glutamine and d-glutamate metabolism, glutathione metabolism, and citrate cycle were associated with LUT treating UC. This study offers a basis for further research on the identification of metabolites, pharmacological mechanism, and clinical applications of LUT on UC.

## Supplementary Material

Supplementary material
