# Supplementary material for: Luteolin alleviates ulcerative colitis in rats via regulating immune response, oxidative stress, and metabolic profiling
Source: Open Med (Wars). 2023 Aug 30;18(1):20230785. doi: 10.1515/med-2023-0785 (PMC10487402; doi:10.1515/med-2023-0785)
Supplement: Supplementary material [file med-2023-0785-sm.pdf]

Supplementary material

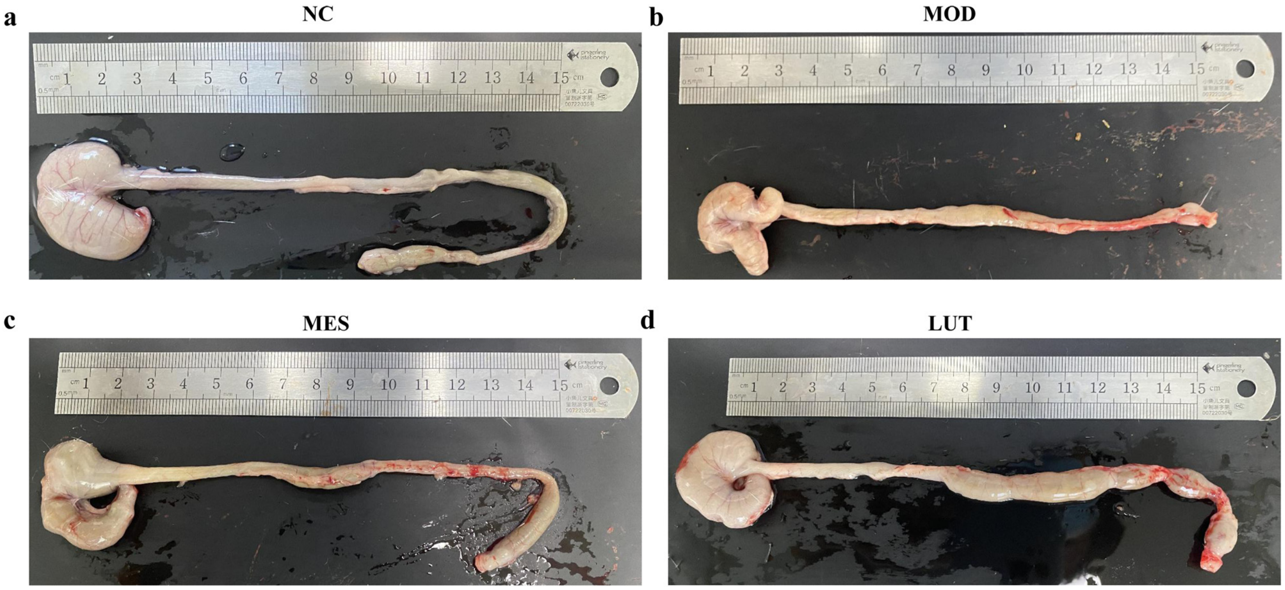

Figure S1: Representative images of colons from each group of rats. Colon lengths of rats in (a) normal control (NC), (b) model (MOD), (c) mesalazine (MES), and (d) luteolin (LUT) group were assayed.

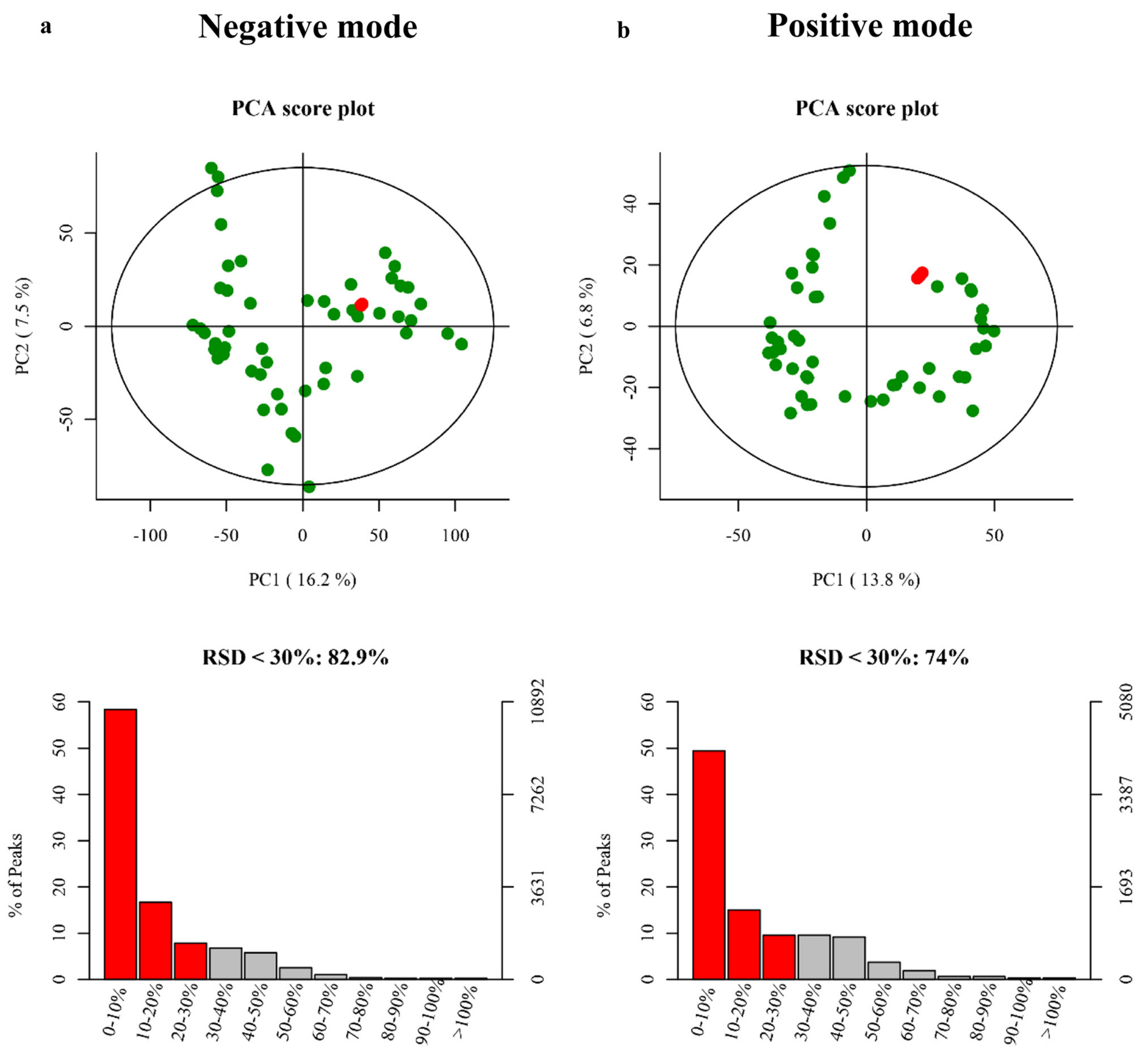

Figure S2: Principal Component Analysis (PCA) score plot and the distribution of relative standard deviation (RSD) of all metabolites in ulcerative colitis (UC) rats. (a) Negative ion mode. (b) Positive ion mode.

Table S1: Body weight of rats in normal control (NC), model (MOD), mesalazine (MES), and luteolin (LUT) group

	Mean weight (g)							
**Group**	**DAY 0**	**DAY 2**	**DAY 4**	**DAY 6**	**DAY 8**	**DAY 10**	**DAY 12**	**DAY 14**
NC	270	281.5	286.5	297.5	308.5	310	316.5	319
MOD	263	265.5	269.5	278	280	269	260.5	245
MES	270.5	271	274.5	282	301.5	299	294	291.5
LUT	260	268	282	286.5	293.5	290	272	272

Table S2: Analysis of potential metabolites of ulcerative colitis rats intervened by luteolin

**Metabolites**	**Normal control vs Model**			**Model vs Luteolin**		
	**log2(FC_NC/MOD)**	**p.value**	**Trend**	**log2(FC_MOD/LUT)**	**p.value**	**Trend**
9-OxoODE	2.5588	0.000449	↑	−1.334	0.023741	↓
(R)-3-Hydroxybutyric acid	1.7199	0.006728	↑	−1.6484	0.00814	↓
Jasmonic acid	1.6591	0.001182	↑	−0.76809	0.04854	↓
Androsterone	1.2644	0.002174	↑	−1.4027	0.014138	↓
Arachidic acid	1.1188	0.011332	↑	−1.9763	0.001266	↓
Homovanillin	1.1109	0.045589	↑	−1.1749	0.001764	↓
Pyrimidodiazepine	0.93065	0.000862	↑	−0.59774	0.00814	↓
Cysteine-S-sulfate	0.81266	0.036795	↑	−2.0786	0.000636	↓
5-Methylcytosine	0.64881	0.000449	↑	−0.49162	0.004531	↓
17a-Estradiol	0.57005	0.002174	↑	−0.43259	0.004531	↓
Dimethyl sulfone	0.11805	0.018543	↑	−0.34034	0.001266	↓
L-Lactic acid	−0.15183	0.036795	↓	0.23821	0.00814	↑
Pyrrolidonecarboxylic acid	−0.3049	0.000624	↓	0.33566	0.000636	↑
L-Malic acid	−0.41263	0.018543	↓	1.1857	0.000636	↑
L-Glutamine	−0.46708	0.000449	↓	0.14234	0.030383	↑
Isocitric acid	−0.53988	0.003881	↓	1.938	0.000636	↑
6-Methylmercaptopurine	−0.54548	0.036795	↓	2.4042	0.000636	↑
N-Formyl-L-methionine	−0.54794	0.045589	↓	2.328	0.000636	↑
Indoleglycerol phosphate	−0.60004	0.000862	↓	0.43622	0.018398	↑
Dethiobiotin	−0.65804	0.045589	↓	2.871	0.000636	↑
S-Lactoylglutathione	−0.67204	0.014548	↓	1.157	0.001764	↑
3-(3,4-Dihydroxyphenyl)pyruvate	−0.79446	0.008764	↓	2.3275	0.000636	↑
Gluconic acid	−0.8323	0.005129	↓	0.9938	0.003337	↑
Phenylacetic acid	−0.86211	0.001609	↓	1.4821	0.000636	↑
Creatinine	−0.86246	0.023468	↓	2.6401	0.003337	↑
2-Isopropyl-3-oxosuccinate	−0.87832	0.006728	↓	1.9603	0.000636	↑
Carnosine	−0.97599	0.045589	↓	1.2482	0.023741	↑
O-Phosphoethanolamine	−1.6089	0.001182	↓	1.5117	0.003337	↑
Deoxyuridine	−1.654	0.006728	↓	1.2102	0.006099	↑
Pimelic acid	−1.6917	0.005129	↓	2.2556	0.000636	↑
6-Ketoprostaglandin E1	−2.2386	0.011332	↓	3.1844	0.002437	↑

Table S3: Main metabolic pathways between the model group and the luteolin group

Metabolic pathway	Raw p	−LOG(p)	Impact	compounds
D-Glutamine and D-glutamate metabolism	0.005129	5.2728	1	L-Glutamic acid; L-Glutamine; Oxoglutaric acid
Alanine, aspartate and glutamate metabolism	0.000465	7.6742	0.65294	L-Glutamic acid; L-Glutamine; N-Acetyl-L-aspartic acid; Succinic acid semialdehyde; gamma-Aminobutyric acid; Fumaric acid;Pyruvic acid; Oxoglutaric acid
Glutathione metabolism	0.017753	4.0312	0.55724	Glutathione; Oxidized glutathione; Pyroglutamic acid; L-Glutamic acid; gamma-Glutamylcysteine; Putrescine
beta-Alanine metabolism	0.21067	1.5575	0.44444	gamma-Aminobutyric acid; beta-Alanine; Anserine
Pyruvate metabolism	0.10691	2.2358	0.3622	S-Lactoylglutathione; L-Lactic acid; L-Malic acid; Pyruvic acid
Arginine and proline metabolism	0.002577	5.9611	0.32961	Pyrroline hydroxycarboxylic acid; gamma-Aminobutyric acid; L-Glutamic acid; L-Glutamine; N-Acetylornithine; L-Glutamic gamma-semialdehyde; Putrescine; 4-Acetamidobutanoic acid; Fumaric acid; N-Acetylglutamic acid
Citrate cycle (TCA cycle)	0.021511	3.8392	0.25323	Isocitric acid; Oxoglutaric acid; L-Malic acid; Pyruvic acid; Fumaric acid
